# Distinguishing benign from malignant thyroid nodules via virtual biopsy: a study on using quantitative parameters and classical radiomics features from dual-energy CT imaging

**DOI:** 10.1186/s12885-025-15261-y

**Published:** 2025-11-25

**Authors:** Jian He, Changyu Du, Mengting Hu, Jingyi Zhang, Qiye Cheng, Yijun Liu, Jianying Li, Jiageng Shen

**Affiliations:** 1 Department of Radiology, First Affiliated Hospital of Dalian Medical University, Xigang District, Lianhe Road, No.193, Dalian, China; 2 CT Research, GE Healthcare, Dalian, China

**Keywords:** Thyroid nodule, Radiomics, Spectral computed tomography, Benign and malignant, Differential

## Abstract

**Background:**

To evaluate the predictive value of dual-energy computed tomography (DECT)-based quantitative parameters and radiomics models for the preoperative differentiation between benign and malignant thyroid nodules, and to compare their performance with radiologists’ interpretations.

**Methods:**

A retrospective analysis was conducted on 215 patients who underwent contrast-enhanced DECT of the thyroid, with pathological outcomes for thyroid nodules obtained. Patients were randomly assigned to training and testing groups in a 7:3 ratio. The images were evaluated by radiologists. Quantitative parameters derived from DECT were identified through univariate and multivariate logistic regression analyses to construct a DECT model. Radiomics features were extracted from the 40, 70, and 100 keV virtual monochromatic images, as well as iodine-based material-decomposition (IMD) images in the arterial phase (AP) and venous phase (VP) to develop radiomics models from both individual and combined images using a support vector machine (SVM), with the optimal performing model selected as the final radiomics model. Subsequently, a fusion model combining DECT parameters and the radiomics model was established. The diagnostic performances of the radiologist, DECT, radiomics and fusion models were evaluated using receiver operating characteristic (ROC) curves, and clinical usefulness was assessed through decision curve analysis (DCA).

**Results:**

The normalized iodine concentration (NIC) in DECT emerged as an independent factor for the preoperative differentiation between benign and malignant thyroid nodules. The multi-image radiomics model demonstrated excellent predictive performance in the test cohort, achieving an area under the curve (AUC) of 0.966 and was selected as the final model among the radiomics models. The ROC curves indicate that the radiomics model outperformed the radiologist model in predicting the thyroid nature in both the training (0.990 vs. 0.746) and test cohorts (0.966 vs. 0.697) (all *p* < 0.05). The diagnostic efficacy of the fusion model showed slight improvement over the radiomics model, although this difference was not statistically significant. Furthermore, the fusion model performed exceptionally well in DCA.

**Conclusions:**

The fusion model, which combined the NIC and the optimal multi-image radiomics model, demonstrates strong diagnostic capability in predicting benign and malignant thyroid nodules.

**Supplementary Information:**

The online version contains supplementary material available at 10.1186/s12885-025-15261-y.

## Introduction

 The prevalence of thyroid nodules among the general population is approximately 67%. The malignancy rate of these nodules varies between 10% and 15% [1]. Accurate assessment of the characteristics of these nodules is essential for formulating appropriate treatment plans. Typically, benign nodules are monitored or treated using less invasive methods, while malignant nodules generally require more intensive interventions, including surgical removal and radioactive iodine therapy. Therefore, distinguishing between benign and malignant thyroid nodules is crucial to avoid unnecessary procedures for benign conditions and to ensure timely treatment for cancerous cases [2].

According to the American Thyroid Association guidelines, ultrasound (US) is the first-line imaging modality for the detection of thyroid nodules. However, its diagnostic accuracy is highly dependent on operator expertise and requires specialized training. Furthermore, it has limitations in the overlap of US features between benign and malignant nodules, as well as the inability to fully depict the three-dimensional anatomical relationships surrounding the thyroid gland [3, 4]. Magnetic resonance imaging (MRI) provides excellent soft tissue contrast, allowing for superior visualization of spatial relationships between thyroid lesions and adjacent anatomical structures [5]. Nevertheless, MRI is a time-intensive procedure and is contraindicated in patients with claustrophobia or implanted cardiac devices such as pacemakers. Additionally, it has limited sensitivity in detecting calcifications, which are critical imaging markers for distinguishing between benign and malignant nodules [6]. Computed tomography (CT) serves as an important supplement to US [7], enabling nodule identification, preoperative localization, diagnostic evaluation, and assessment of treatment response [8]. Compared with conventional CT, dual-energy CT (DECT) acquires data at two distinct X-ray tube voltages, yielding virtual monoenergetic images (VMIs) and material decomposition images [9–12]. This advanced technique facilitates objective lesion characterization and provides detailed three-dimensional anatomical information, thereby supporting comprehensive evaluation of thyroid pathology [13]. The generation of material decomposition images can better reflect tissue and lesion characteristics, with quantitative iodine parameters determined from iodine-based material decomposition (IMD) images regarded as promising tools for distinguishing malignant from benign tumors [13–15]. In addition, spectral curves and effective atomic number (Z_eff_) in DECT assist in differentiating benign and malignant tumors, histological types, and degrees of differentiation [16–19]. Liu et al. [20] confirmed that DECT quantitative assessment has higher accuracy than conventional CT and can directly differentiate between metastatic and non-metastatic lymph nodes. Compared to DECT quantitative parameters, radiomics can further assess tumor heterogeneity, which is highly correlated with tumor malignancy. Radiomics enables the extraction of high-throughput quantitative features from digital medical images, which can reveal underlying pathophysiological mechanisms but are often not easily discernible to the naked eye of radiologists. It has been proven to be an effective tool for enhancing the accuracy of screening, diagnosis, prognosis, and prediction [6, 21, 22]. Zhou et al. [23] have shown that radiomics based on iodine maps can effectively predict lymph node metastasis in thyroid cancer. However, to the best of our knowledge, few studies have explored the comprehensive application value of DECT material decomposition images, VMIs, and radiomics in differentiating benign from malignant thyroid nodules.

Therefore, this study aims to develop an optimal DECT radiomics model for evaluating the nature of thyroid nodules and to explore the clinical significance of combining quantitative parameters with radiomics features in DECT imaging for the preoperative prediction of benign and malignant thyroid nodules.

## Materials and methods

### Study population

**Fig. 1:**
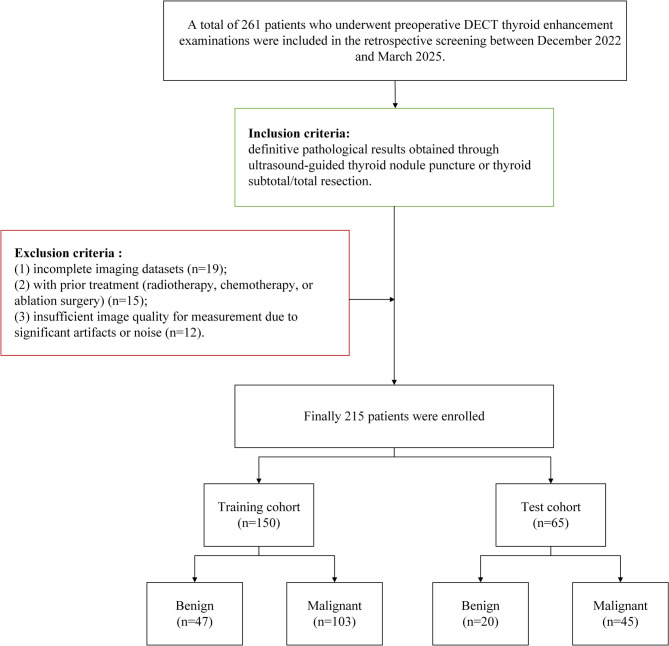
The flow chart of the study population

This retrospective study was approved by the Ethics Committee of our hospital (IRB No. PJ-KS-KY-2024-54), which waived the requirement for informed consent. The study was conducted in accordance with the Declaration of Helsinki. A total of 261 patients who underwent preoperative DECT thyroid enhancement examinations between December 2022 and March 2025 were included in the retrospective screening. The inclusion criteria encompassed definitive pathological results obtained through ultrasound-guided thyroid nodule puncture or thyroid subtotal/total resection. The exclusion criteria included: (1) incomplete imaging datasets (*n* = 19); (2) with prior treatment (radiotherapy, chemotherapy, or ablation surgery) (*n* = 15); and (3) insufficient image quality for measurement due to significant artifacts or noise (*n* = 12). Ultimately, 215 patients were included in the study. These patients were randomly divided into a training cohort (150 cases) and a test cohort (65 cases) in a 7:3 ratio. The flowchart of the study population is illustrated in Fig. 1.

### Image acquisition

A 256-row DECT scanner (Revolution CT, GE HealthCare, Milwaukee, WI, USA) was used for image acquisition covering from the cranial base to the apex of the aortic arch. The acquisition specifications included spectral imaging via Gemstone Spectral Imaging (GSI) mode with instantaneous tube voltage alternation (80/140 kVp), automated tube current modulation (GSI Assist; target noise index: 10), detector coverage of 80 mm, a helical pitch ratio of 0.992:1, and a gantry rotation time of 0.6 s. A nonionic iodinated contrast medium (Ioversol, 350 mg I/mL; Hengrui Pharma, China) was administered intravenously through forearm access at a volume of 70 mL (3.0–4.0 mL/s flow rate), followed immediately by a saline flush matching the contrast delivery rate to standardize a total injection duration of 30 s. The scan delay time was 25 s for the arterial phase (AP) and 50 s for the venous phase (VP). All images were reconstructed using the adaptive statistical iterative reconstruction V (ASIR-V) algorithm with 40% intensity and a slice thickness of 2.5 mm.

### Quantitative parameter measurement

Spectral images of AP and VP were generated using the Advantage Workstation 4.7, a dedicated postprocessing software (GE Healthcare), to reconstruct VMIs at three distinct energy levels (40, 70, and 100 keV), as well as IMD and Z_eff_ images. Quantitative parameters were assessed on three adjacent slices encompassing the largest area and significant portions of the primary lesion while excluding cystic areas, calcifications, and necrosis. The quantitative parameters derived from DECT images, including both AP and VP, comprised the following: (I) iodine concentration (IC) and Z_eff_, measured from the IMD and Z_eff_ maps, respectively; (II) normalized iodine concentration (NIC) calculated as NIC = IC of the lesion/IC of the carotid; and (III) the slope of the spectral Hounsfield unit (HU) curve (λ_HU_), defined as λ_HU_ = (CT value at 40 keV - CT value at 100 keV)/(100 − 40).

### Images segmentation

**Fig. 2:**
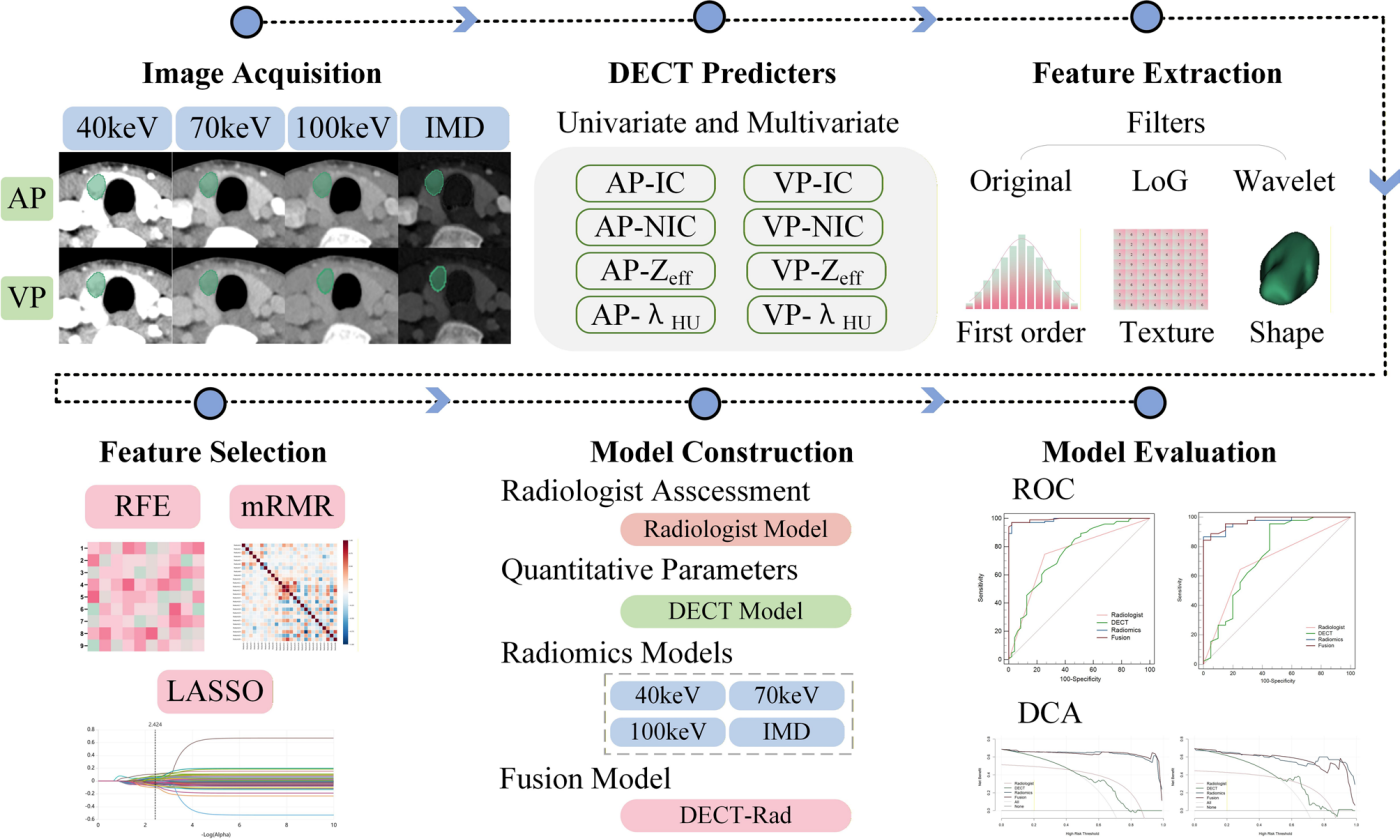
The workflow of the study

The images at 40, 70, and 100 keV, along with the IMD images in AP and VP, were imported into the uAI Research Portal V1.1 software developed by Shanghai United Imaging Intelligence Co., Ltd. A radiologist with 5 years of experience in imaging head and neck tumors delineated the lesion layer by layer on the 40 keV axial images until the entire lesion was delineated. The radiologist was unaware of the clinical data or pathological results during the delineation process. To ensure accuracy, a senior radiologist with 15 years of experience in head and neck imaging verified and corrected all segmentation masks. Discrepancies were resolved through consensus-based discussions. In cases with multiple lesions, only the largest lesion was selected for delineation. Subsequently, the resulting volume of interest (VOI) was copied to other image types. A workflow of this study is shown in Fig. 2.

### Feature extraction

A standardized pre-processing pipeline was implemented to reduce variability in spatial resolution and intensity across imaging data. First, all images were resampled to an isotropic resolution of 1 × 1 × 1 mm using B-spline interpolation. Subsequently, intensity normalization was performed via Z-score standardization, followed by gray-level discretization into 25 bins. Radiomic features were extracted from each preprocessed image using the uAI Research Portal V1.1 software. The Pyradiomics software package (version 3.0.1), available at https://pypi.org/project/pyradiomics/, was used to extract 1,184 radiomic features from both the arterial phase (AP) and venous phase (VP) images at 40, 70, and 100 keV energy levels, as well as from the IMD images, using three filters: the original, wavelet, and Laplacian of Gaussian (LoG) filters. These radiomic features were categorized into the following classes: (1) first-order features; (2) shape features; and (3) texture features, which include gray level co-occurrence matrix (GLCM) features, gray level size zone matrix (GLSZM) features, gray level run length matrix (GLRLM) features, gray level difference matrix (GLDM) features, and neighboring gray tone difference matrix (NGTDM) features. One month later, 30 samples were randomly selected, and two radiologists performed independent segmentation once again. Subsequently, all radiomics features extracted from the VOI segmentations conducted by the two radiologists were assessed for intra-observer and inter-observer reproducibility using the intraclass correlation coefficient (ICC), with a value of ≥ 0.75 deemed robust.

### Feature selection

Initially, all radiomics features were standardized using the Z-score normalization method. Subsequently, a sequential feature selection strategy was employed, incorporating Recursive Feature Elimination (RFE), Minimum Redundancy Maximum Relevance (mRMR), and Least Absolute Shrinkage and Selection Operator (LASSO). RFE iteratively removes the least contributive radiomics features for distinguishing between benign and malignant thyroid nodules, dynamically optimizing the feature subset to enhance the model’s diagnostic performance. Meanwhile, mRMR identifies feature combinations that are highly correlated with tumor malignancy while minimizing redundancy, thereby improving the model’s discriminative specificity for thyroid lesions. LASSO employs L1 regularization to reduce the regression coefficients of non-essential features to zero, effectively isolating core radiomics indicators with robust diagnostic utility.

### Models building

#### Radiologist model

All CT images of thyroid nodules in the training and test cohorts were independently reviewed by two radiologists, one with 5 years and the other with 15 years of experience in head and neck imaging. They evaluated qualitative CT radiological features strongly associated with benign and malignant thyroid nodules, including location, lesion morphology, cystic changes, microcalcifications, edge interruption signs, and blurring after enhancement. Each conducted three assessments at one-week intervals, reviewing the images in a random order. If the diagnoses from the three assessments were inconsistent, the consistent diagnosis in any two assessments was deemed the final interpretation. In cases of discrepancies between the two readers, a third radiologist with 20 years of experience in the same field was consulted to review the images and provide a definitive adjudication. Throughout the study, the observers remained completely blinded to both the study design and the corresponding clinicopathological findings.

#### DECT model

Initially, the differences in DECT quantitative parameters between benign and malignant nodules were assessed using univariate analysis. Subsequently, quantitative parameters with a p-value of less than 0.05 from the univariate analysis were included in a multivariate analysis to identify independent predictors for distinguishing benign from malignant thyroid nodules. Logistic regression (LR) was employed to construct the DECT model.

#### Radiomics model

Support Vector Machines (SVM) were utilized to construct four radiomics models using the individual VMIs at 40, 70, and 100 keV, as well as the IMD images combining both AP and VP features. Subsequently, the features from these three VMI sets and one IMD image in both AP and VP, totaling eight VOIs, were integrated, and SVM was employed to establish a multi-image model. A total of 5 radiomics models (based on the 40, 70, 100 VMIs, IMD images, and multi-images, all combining both AP and VP features) were ultimately created, with the aim of identifying the optimal radiomics model within both the training and test sets. The hyperparameters of the Support Vector Machine (SVM) classifier are detailed in the supplementary material.

#### Fusion model

The optimal radiomics model was fused with independent predictors from the DECT model using forward selection logistic regression to construct a fusion model for predicting benign and malignant thyroid nodules. To achieve balanced samples, we implemented the SMOTE oversampling technique, which synthesizes new minority class samples to enhance the dataset’s generalization and accuracy.

#### Model evaluation

The ROC analyses were performed to visualize model performance, with comprehensive evaluation of diagnostic effectiveness conducted through multiple metrics such as sensitivity, specificity, accuracy, precision, and the area under the curve (AUC). Furthermore, DCA was implemented to quantify clinical applicability by determining net benefit thresholds across different probability ranges.

### Statistical analysis

Statistical analyses were conducted using three computational platforms: MedCalc (v22.009; MedCalc Software Ltd, Belgium), R statistical environment (v4.2.1), and SPSS (v27.0; IBM Corp, USA). Continuous variables underwent preliminary normality evaluation through the Shapiro-Wilk normality test. Parametric measures were summarized using mean values with standard deviations, while non-normally distributed data were characterized by median values and interquartile ranges. Comparative analyses between groups utilized appropriate statistical tests, including Student’s t-tests for parametric data, Mann-Whitney U tests for non-parametric comparisons, and χ² tests for categorical variables. Diagnostic performance metrics encompassing sensitivity, specificity, accuracy, precision, and AUC were calculated to assess the model’s diagnostic efficacy. A comparative analysis of receiver operating characteristic curves was performed using the DeLong method. The Benjamini-Hochberg false discovery rate (FDR) procedure was employed for multiple comparisons, with statistical significance defined as an FDR-adjusted P-value (q-value) < 0.05. Analytical computations for diagnostic accuracy metrics were specifically executed within the R programming ecosystem to align with contemporary methodological standards in clinical statistics.

## Results

### General information

This study encompassed a total of 215 patients. There were 67 cases of benign nodules, comprising 51 cases of nodular goiter, 8 cases of follicular adenoma, 6 cases of necrotizing granulomatous lesions, and 2 cases of eosinophilic adenoma. Additionally, there were 148 cases of malignant lesions, which included 144 cases of papillary carcinoma, 2 cases of medullary carcinoma, and 2 cases of minimally invasive follicular carcinoma.

### Clinical characteristics and radiological features

The clinical characteristics and radiological features of patients in the training and test groups are summarized in Table S1. No statistically significant differences were found across all indicators between the training and test sets. In both the training and test sets, there were no statistically significant differences in gender or lesion location between benign and malignant nodules; however, statistically significant differences were observed in age, lesion morphology, cystic changes, microcalcifications, the interrupted margin sign, and post-enhancement blurring.

### Radiologist model

In the training dataset, the radiologist model achieved an AUC, accuracy, sensitivity, and specificity of 0.746, 0.747, 0.748, and 0.745, respectively. In the test dataset, the model achieved an AUC, accuracy, sensitivity, and specificity of 0.697, 0.677, 0.644 and 0.750, respectively.

### DECT model

The DECT quantitative parameters (AP-IC, AP-NIC, AP-Z_eff_, AP-λ_HU_, VP-IC, VP-NIC, VP-Z_eff_, VP-λ_HU_) were summarized in Table 1, all except VP-λ_HU_ were identified as significant risk factors for predicting benign and malignant thyroid nodules. Further multivariate logistic regression analysis revealed that AP-NIC (OR, 1181.216; 95% CI: 10.676–130744.459.676.459; *P*<0.05) was the only independent predictor (Table 2). Therefore, AP-NIC was used to construct the DECT model.

**Table 1:** Comparison of DECT quantitative parameters between benign and malignant nodules

Variables	Training cohort (*n* = 150)			Test cohort (*n* = 65)		
	Benign(*n* = 47)	Malignant(*n* = 103)	*P* value	Benign(*n* = 20)	Malignant(*n* = 45)	*P* value
AP-IC(mg/mL)	39.67(26.37,47.98)	49.85(40.94,57.38)	<0.001	36.89(29.33,54.02)	46.72(40.91,54.92)	0.129
AP-NIC	0.30(0.21,0.37)	0.40(0.34,0.50)	<0.001	0.29(0.22,0.40)	0.41(0.35,0.50)	0.02
AP-Z_eff_	9.67(9.08,9.99)	10.06(9.66,10.32)	<0.001	9.77(9.25,10.43)	9.95(9.70,10.24)	0.366
AP-λ_HU_	4.72(3.16,5.80)	5.74(4.55,6.81)	0.001	4.42(3.47,7.95)	5.41(4.79,6.67)	0.485
VP-IC(mg/mL)	31.14(24.68,38.94)	34.29(29.39,39.98)	0.036	34.52(27.12,42.50)	32.03(26.42,35.28)	0.681
VP-NIC	0.80(0.62,0.97)	0.87(0.73,1.02)	0.03	0.88(0.66,1.07)	0.82(0.69,0.95)	0.424
VP-Z_eff_	9.30(8.98,9.63)	9.41(9.21,9.61)	0.033	9.42(9.11,9.76)	9.33(9.07,9.47)	0.532
VP-λ_HU_	3.73(2.97,4.69)	4.02(3.24,4.60)	0.131	4.11(3.26,5.12)	3.87(3.35,4.24)	0.269

*AP* Arterial phase, *VP* Venous phase, *IC* Iodine concentration, *NIC* Normalized iodine concentration, *Z*_eff_ Effective atomic number, λ_HU_ the slope of spectral HU curve

**Table 2:** Independent factors for predicting benign and malignant thyroid nodules

Variables	Univariate analysis			Multivariate analysis		
	OR	95% CI	*P* value	OR	95% CI	*P* value
AP-IC(mg/mL)	1.020	1.013–1.026	0.000	1181.216	10.676–130744.459.676.459	0.013
AP-NIC	1.025	1.016–1.034	0.000			
AP-Z_eff_	1.083	1.052–1.115	0.000			
AP-λ_HU_	1.085	1.052–1.119	0.000			
VP-IC(mg/mL)	1.174	1.113–1.239	0.000			
VP-NIC	1.222	1.137–1.314	0.000			
VP-Z_eff_	2.560	1.835–3.572	0.000			
VP-λ_HU_	12.394	5.692–26.977	0.000			

*OR* Odds ratio, *CI* Confidence interval, *IC* Iodine concentration, *NIC* Normalized iodine concentration, *FC* Fat water concentration, *Z*_eff_ Effective atomic number

### Radiomics model

Initially, a total of 2368, 2368, 2368, 2368, 9472 radiomics features were utilized for feature selection. After this process, 26, 22, 18, 18, and 32 features were identified for constructing the 40 keV, 70 keV, 100 keV, IMD, and multi-image models, respectively, using SVM classifiers. The specific features are depicted in Supplementary Figure S1. The final features selected for the multi-image model are illustrated in Table S2. The diagnostic performances of radiomics models in the training and test cohorts are shown in Figure S2 and Table 3. The diagnostic performance based on multi-image was the best in differentiating benign and malignant thyroid nodules in the test cohorts (AUC = 0.966), with a sensitivity and specificity of 0.933 and 0.850, respectively. Moreover, DCA demonstrated that the multi-image model could provide greater net benefits compared to other radiomics models (Figure S3). Therefore, we selected the multi-image radiomics model as the final radiomics model.

**Table 3:** The diagnostic performance of radiomics models

Cohort	Model	AUC (95% CI)	SEN	SPE	ACC	PRE
Training	40	0.951(0.903,0.979)	0.893	0.915	0.900	0.958
	70	0.967(0.925,0.989)	0.913	0.936	0.920	0.969
	100	0.946(0.897,0.976)	0.845	0.957	0.880	0.978
	IMD	0.938(0.887,0.971)	0.942	0.915	0.933	0.960
	Multi-image	0.990(0.957,0.999)	0.971	0.979	0.973	0.990
Test	40	0.903(0.804,0.963)	0.733	0.950	0.800	0.971
	70	0.816(0.700,0.901)	0.889	0.650	0.815	0.851
	100	0.803(0.686,0.892)	0.800	0.750	0.785	0.878
	IMD	0.901(0.802,0.961)	0.956	0.750	0.892	0.896
	Multi-image	0.966(0.888,0.995)	0.867	1.000	0.908	1.000

*AUC* Area under the curve, *CI* Confidence interval, *SEN* Sensitivity, *SPE* specificity, *ACC* Accuracy, *PRE* Precision

### Fusion model

The multi-image radiomics model was integrated with AP-NIC to construct a fusion model. The results showed that in the test set, the AUC value of the fusion model achieving the highest performance (AUC = 0.974).

### Model comparison

**Fig. 3:**
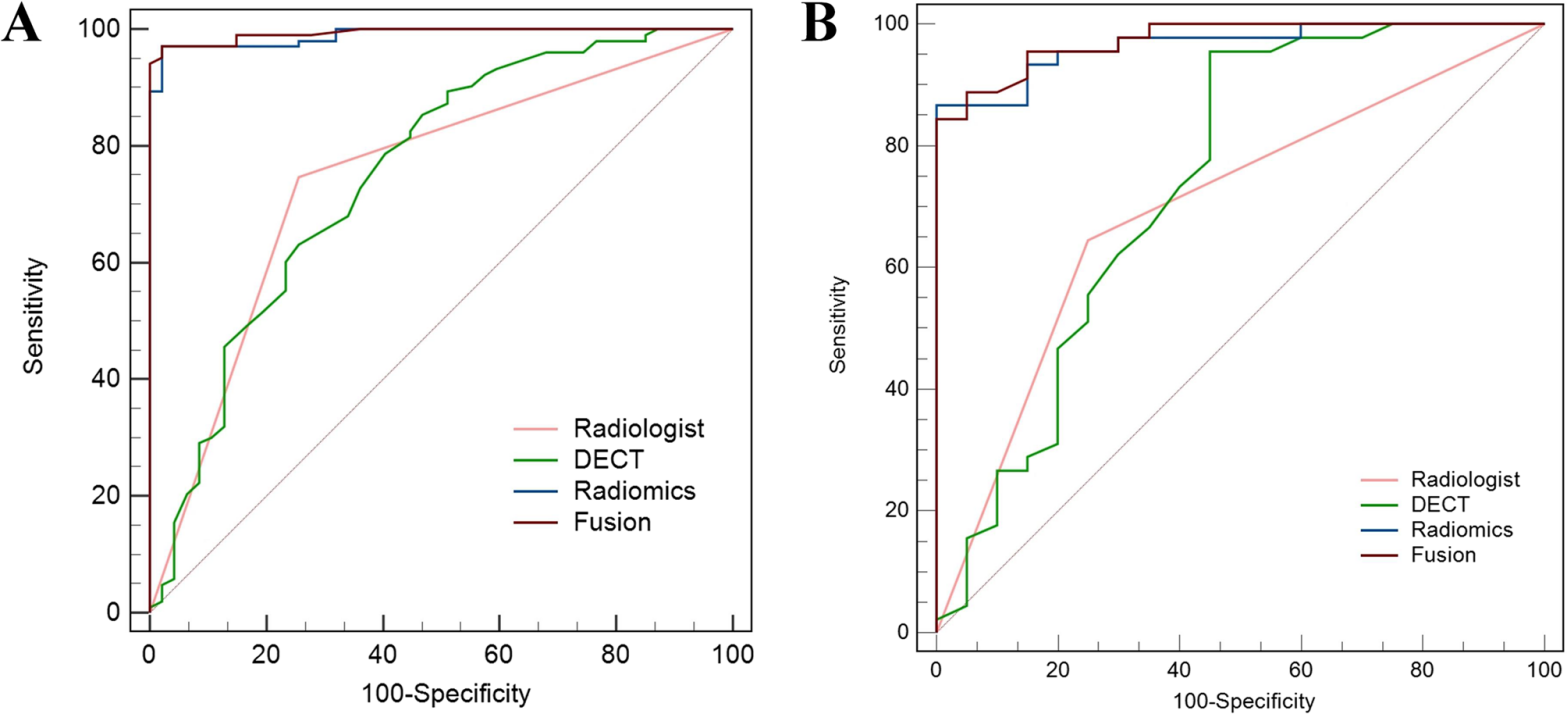
Receiver operating characteristic curves for radiologist, DECT, radiomics, and fusion models in training (**A**) and test cohorts (**B**)

The ROC curves of the four models in differentiating benign and malignant thyroid nodules are shown in Fig. 3, while the results of the DeLong test comparing these models’ performances are displayed in Table 4.

**Table 4:** DeLong test for the comparison of AUC values between different models

Cohort	Model comparison	Z	*P*	Q
Training	Radiologist vs. DECT	0.123	0.902	0.902
	Radiologist vs. Radiomics	6.229	< 0.001	< 0.001
	Radiologist vs. Fusion	6.361	< 0.001	< 0.001
	DECT vs. Radiomics	5.191	< 0.001	< 0.001
	DECT vs. Fusion	5.295	< 0.001	< 0.001
	Radiomics vs. Fusion	0.544	0.586	0.703
Test	Radiologist vs. DECT	0.418	0.676	0.732
	Radiologist vs. Radiomics	4.175	< 0.001	0.000
	Radiologist vs. Fusion	4.376	< 0.001	0.000
	DECT vs. Radiomics	2.874	0.004	0.006
	DECT vs. Fusion	3.012	0.003	0.006
	Radiomics vs. Fusion	0.343	0.732	0.732

**Fig. 4:**
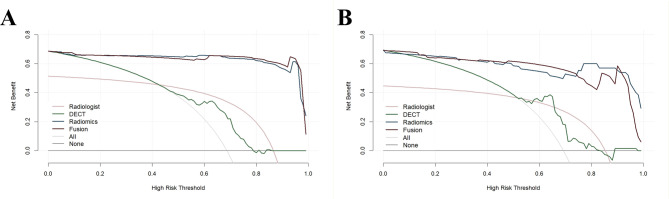
Decision curves for radiologist, DECT, radiomics, and fusion models in training (**A**) and test cohorts (**B**). Fusion model and radiomics model had higher net benefits than DECT model

The ROC curves indicated that the radiomics model outperformed the radiologist model in predicting the thyroid nature in both the training set (0.990 vs. 0.746) and the test set (0.966 vs. 0.697) (all q < 0.05). Furthermore, after integrating AP-NIC, the fusion model demonstrated an incremental improvement in performance (0.974 vs. 0.966 in the test set), although this improvement did not reach statistical significance after FDR adjustment (q > 0.05). Furthermore, after integrating AP-NIC, the fusion model demonstrated an incremental improvement in performance (0.974 vs. 0.966 in the test), although the difference was not statistically significant. DCA demonstrated that across all threshold probability ranges (Fig. 4), the fusion model offered a greater net benefit compared to the all-or-none intervention strategy, thereby reducing unnecessary biopsies.

## Discussion

This study is the first to construct a fusion model that combines radiomics features with quantitative parameters derived from DECT to differentiate between benign and malignant thyroid nodules. The results demonstrated that this fusion model achieved superior performance in both training and test datasets compared to the visual assessments made by radiologists. Furthermore, the performance of the fusion model demonstrated a numerically higher and more consistent AUC compared to the radiomics model, suggesting that DECT quantitative parameters can serve as valuable supplements to radiomic features for the differential diagnosis of benign and malignant thyroid nodules. Our findings indicated that the fusion model holds promise as a non-invasive tool for the preoperative prediction of benign and malignant thyroid nodules.

DECT enhances the capabilities of conventional CT by incorporating energy information, allowing the acquisition of quantitative parameters that cannot be obtained through standard CT imaging, such as the IC, λ_HU_, and Z_eff_. Previous studies have established that these quantitative parameters correlate with the malignancy of thyroid nodules [13, 14, 16–19], prompting us to include them in our research. Our univariate analysis revealed that, with the exception of VP-λ_HU_, all parameters exhibited statistical significance. However, in the multivariate analysis, most variables were excluded, with only AP-NIC emerging as an independent predictor. This may be attributed to the high degree of correlation among the DECT parameters, which are not truly independent but rather represent distinct mathematical transformations derived from the same underlying imaging process. Collectively, these parameters reflect tissue iodine concentration and perfusion characteristics, leading to substantial overlap in diagnostic information and resulting in multicollinearity [24]. Moreover, the AP-NIC for malignant nodules was significantly higher than that for benign nodules, aligning with findings from previous studies [8, 25], which may be attributed to the increased vascularity associated with thyroid cancer.

Radiomics can further explore information related to tumor heterogeneity and leverage high-throughput quantitative features to reflect the blood supply heterogeneity associated with tumor perfusion and permeability. Current research primarily focuses on predicting the nature, metastasis, or recurrence of thyroid conditions using radiomics based on conventional CT images and quantitative parameters derived from DECT [26, 27]. These methodologies have demonstrated superior diagnostic performance compared to clinical characteristics and visual assessments by radiologists. To date, however, few radiomics analyses utilizing DECT have been conducted to differentiate between benign and malignant thyroid nodules. As an advanced iteration of conventional CT, DECT facilitates the generation of IMD images and a range of VMIs. Previous studies utilizing radiomics on IMD to predict lymph node metastasis (LNM) or recurrence in papillary thyroid carcinoma have demonstrated excellent diagnostic performance, highlighting the utility of IMD-based radiomics in predicting the malignancy of thyroid tumors [28, 29]. Furthermore, an increasing number of studies indicate that the substantial additional information obtained from energy-dependent attenuation variations across different tissues can enhance the performance and accuracy of predictive models in radiomic research [30, 31]. Virtual monochromatic images (VMIs) have demonstrated high diagnostic efficacy in differentiating between benign and malignant thyroid nodules [14, 30]. From the perspective of average attenuation, the 70 keV energy level corresponds to a 120 kVp single-energy CT acquisition [32, 33]. Low-energy images (40–70 keV) more accurately reflect tissue enhancement characteristics, while high-energy images (100–140 keV) provide distinctive features of non-enhanced tissues [34, 35]. Consequently, we selected 70 keV as the equivalent of a standard 120 kVp CT acquisition, designating 40 keV as representing low-energy images and 100 keV for high-energy images to establish distinct radiomic models. Building on this premise, we integrated 40, 70, and 100 keV images together with iodine map decomposition (IMD) images into a multi-image analysis, thereby creating a comprehensive lesion imaging dataset that overcomes the limitations of single-energy imaging techniques. Our research findings indicated that in the test set, the AUC and accuracy of the multi-image radiomics analysis both increased to over 90%, surpassing radiomics models based solely on single images. This finding demonstrated that combining images of different energies enriches the input features and provides more comprehensive information compared to single-image datasets, thereby improving the overall diagnostic accuracy of the radiomics analysis. Finally, we combined the radiomics score with the AP-NIC to establish a fusion model for the preoperative prediction of thyroid nodule characteristics. Encouragingly, this fusion model demonstrated superior predictive accuracy compared to both the DECT model and the radiomics model, suggesting that the DECT model has additional value. This is likely due to the stable and robust performance of the AP-NIC, which reflects the iodine concentration within the lesion and specifically indicates the blood flow characteristics of the nodule. Malignant nodules generally exhibit higher iodine concentrations. Furthermore, standardized iodine concentration accounts for the influence of the contrast agent, leading to more accurate outcomes by eliminating the effects of scanning parameters and contrast agent dosage. Thus, by incorporating DECT quantitative parameters, we enhanced the performance of our model. Comparative analysis with radiologists revealed that the diagnostic performance of this fusion model significantly outperformed visual assessments conducted by radiologists (0.974 vs. 0.697 in the test cohort). Furthermore, sensitivity increased from 64% to 84%, effectively addressing the critical issue of low diagnostic sensitivity often encountered among radiologists. Moreover, this fusion model generated net benefits across nearly all threshold probability ranges when predicting the risk outcomes for benign and malignant thyroid conditions.

This study does have several limitations. First, this study utilized a single-center design with a single scanner model and conducted a retrospective analysis based on a relatively small sample size. Future research should incorporate large-scale, multicenter, multivendor prospective studies to validate and generalize the findings. Second, this study relied on manual tumor segmentation, which is inherently susceptible to inter-observer variability and compromises the reproducibility of radiomic features. To enhance clinical applicability, the development of semi-automated or fully automated segmentation methods is essential. Third, the dataset used in this study exhibits class imbalance. Although we employed the SMOTE to mitigate potential model bias toward the majority class, future research requires more balanced samples for further validation. Fourth, patients who had received preoperative treatments such as radiotherapy, chemotherapy, or ablation procedures were excluded, which may lead to selection bias. Finally, the results were confined to the DECT scanning protocol.

In conclusion, the multi-image radiomics model surpasses both single-energy (40/70/100 keV) and IMD models in differentiating benign from malignant thyroid nodules. The fusion model, which integrates radiomics features with AP-NIC, shows a slight improvement in predicting the nature of thyroid nodules compared to both DECT and radiomics models, while significantly outperforming the diagnostic performance of radiologists’ visual assessments. This fusion model serves as an effective tool for predicting thyroid nodule characteristics, thereby offering valuable support for clinical decision-making.

## Supplementary Information

Supplementary Material 1.

## Data Availability

The datasets used and/or analyzed during the current study are available from the corresponding author on reasonable request.

## Abbreviations

DECT: Dual-energy computed tomography
AP: Arterial phase
VP: Venous phase
IMD: Iodine-based material-decomposition
SVM: Support vector machine
ROC: Receiver operating characteristic
DCA: Decision curve analysis
NIC: Normalized iodine concentration
AUC: Area under the curve
US: Ultrasonography
CT: Computed tomography
VMIs: Virtual monoenergetic images
Z_eff_: Effective atomic number
IRB: Institutional Review Board
GSI: Gemstone Spectral Imaging
ASIR-V: Adaptive statistical iterative reconstruction V
IC: Iodine concentration
HU: Hounsfield unit, λ_HU_ spectral HU curve
VOI: Volume of interest
LoG: Laplacian of Gaussian
GLCM: Gray level co-occurrence matrix
GLSZM: Gray level size zone matrix
GLRLM: Gray level run length matrix
GLDM: Gray level difference matrix
NGTDM: Neighboring gray tone difference matrix
RFE: Recursive Feature Elimination
mRMR: Minimum Redundancy Maximum Relevance
LASSO: Least Absolute Shrinkage and Selection Operator
LR: Logistic regression

## References

[1] Durante C, Grani G, Lamartina L, Filetti S, Mandel SJ, Cooper DS. The diagnosis and management of thyroid nodules. JAMA. 2018;319(9):914. 29509871 10.1001/jama.2018.0898

[2] Oczko-Wojciechowska M, Kotecka-Blicharz A, Krajewska J, Rusinek D, Barczyński M, Jarząb B, et al. European perspective on the use of molecular tests in the diagnosis and therapy of thyroid neoplasms. Gland Surg. 2020;9(Suppl 2):S69-76. 32175247 10.21037/gs.2019.10.26 PMC7044080

[3] Richman DM, Benson CB, Doubilet PM, Wassner AJ, Asch E, Cherella CE, Smith JR, Frates MC. Assessment of American college of radiology thyroid imaging reporting and data system (TI-RADS) for pediatric thyroid nodules. Radiology. 2020;294(2):415–20. 31821121 10.1148/radiol.2019191326

[4] Tappouni RR, Itri JN, McQueen TS, Lalwani N, Ou JJ. ACR TI-RADS: pitfalls, solutions, and future directions. Radiographics. 2019;39(7):2040–52. 31603734 10.1148/rg.2019190026

[5] Kato H, Kanematsu M, Kato Z, Teramoto T, Mizuta K, Aoki M, et al. Necrotic cervical nodes: usefulness of diffusion-weighted MR imaging in the differentiation of suppurative lymphadenitis from malignancy. Eur J Radiol. 2013;82(1):e28-35. 22954412 10.1016/j.ejrad.2012.08.014

[6] Zhang L, Li Y, Chen Z, Dai X, Gao H, Chen Y. Diagnostic performance of dual-energy computed tomography (DECT) quantitative parameters for detecting metastatic cervical lymph nodes in patients with papillary thyroid cancer: a systematic review and meta-analysis. Eur J Radiol. 2025. 10.1016/j.ejrad.2025.111917. 39778378 10.1016/j.ejrad.2025.111917

[7] Mou Y, Han X, Li J, Yu P, Wang C, Song Z, et al. Development and validation of a computed tomography-based radiomics nomogram for the preoperative prediction of central lymph node metastasis in papillary thyroid microcarcinoma. Acad Radiol. 2024;31(5):1805–17. 38071100 10.1016/j.acra.2023.11.030

[8] Jiang L, Liu D, Long L, Chen J, Lan X, Zhang J. Dual-source dual-energy computed tomography-derived quantitative parameters combined with machine learning for the differential diagnosis of benign and malignant thyroid nodules. Quant Imaging Med Surg. 2022;12(2):967–78. 35111598 10.21037/qims-21-501 PMC8739151

[9] Geng D, Zhou Y, Su G-Y, Si Y, Shen M-P, Xu X-Q, et al. Influence of sex, age and thyroid function indices on dual-energy computed tomography-derived quantitative parameters of thyroid in patients with or without Hashimoto’s thyroiditis. BMC Med Imaging. 2023. 10.1186/s12880-023-00983-x. 36740672 10.1186/s12880-023-00983-x PMC9901076

[10] Zhao W, Shen S, Ke T, Jiang J, Wang Y, Xie X, et al. Clinical value of dual-energy CT for predicting occult metastasis in central neck lymph nodes of papillary thyroid carcinoma. Eur Radiol. 2023;34(1):16–25. 37526667 10.1007/s00330-023-10004-8

[11] Zhang H, Zhang L, Long J, Zhang H, Sun X, Zhang S, et al. Image quality improvement in head and neck angiography based on dual-energy CT and deep learning. BMC Med Imaging. 2025;25(1):115. 40211222 10.1186/s12880-025-01659-4 PMC11987473

[12] Chen J, Zhang F, Wu S, Liu D, Yang L, Li M, et al. Predictive value of high-risk esophageal varices in cirrhosis based on dual-energy CT combined with clinical and serologic features. BMC Med Imaging. 2025;25(1):137. 40281459 10.1186/s12880-025-01681-6 PMC12032664

[13] Ren X, Song Z, Zhang D, Li X, Huang J, Liu Q, et al. Differentiation of benign and malignant lesions in Bethesda III and IV thyroid nodules via dual-energy computed tomography quantitative parameters and morphologic features. Quant Imaging Med Surg. 2024;14(7):4567–78. 39022257 10.21037/qims-23-1511 PMC11250302

[14] Song Z, Li Q, Zhang D, Li X, Yu J, Liu Q, et al. Nomogram based on spectral CT quantitative parameters and typical radiological features for distinguishing benign from malignant thyroid micro-nodules. Cancer Imaging. 2023;23(1):13. 36703218 10.1186/s40644-023-00525-2 PMC9878766

[15] Li Z, Wang N, Bing X, Li Y, Yao J, Li R, et al. The value of a dual-energy CT iodine map radiomics model for the prediction of collagen fiber content in the CcRCC tumor microenvironment. BMC Med Imaging. 2023;23(1):186. 37968599 10.1186/s12880-023-01127-x PMC10648380

[16] Karino T, Ohira S, Kanayama N, Wada K, Ikawa T, Nitta Y, et al. Determination of optimal virtual monochromatic energy level for target delineation of brain metastases in radiosurgery using dual-energy CT. Br J Radiol. 2020;93(1106):20180850. 31825643 10.1259/bjr.20180850 PMC7055442

[17] Patel BN, Boltyenkov AT, Martinez MG, Mastrodicasa D, Marin D, Jeffrey RB, et al. Cost-effectiveness of dual-energy CT versus multiphasic single-energy CT and MRI for characterization of incidental indeterminate renal lesions. Abdom Radiol. 2020;45(6):1896–906. 10.1007/s00261-019-02380-x 31894384

[18] Volterrani L, Gentili F, Fausto A, Pelini V, Megha T, Sardanelli F, et al. Dual-energy CT for locoregional staging of breast cancer: preliminary results. AJR Am J Roentgenol. 2020;214(3):707–14. 31939699 10.2214/AJR.18.20953

[19] Wang X, Liu D, Zeng X, Jiang S, Li L, Yu T, et al. Dual-energy CT quantitative parameters for the differentiation of benign from malignant lesions and the prediction of histopathological and molecular subtypes in breast cancer. Quant Imaging Med Surg. 2021;11(5):1946–57. 33936977 10.21037/qims-20-825 PMC8047348

[20] Liu X, Ouyang D, Li H, Zhang R, Lv Y, Yang A, et al. Papillary thyroid cancer: dual-energy spectral CT quantitative parameters for preoperative diagnosis of metastasis to the cervical lymph nodes. Radiology. 2015;275(1):167–76. 25521777 10.1148/radiol.14140481

[21] Zheng S, Liu J, Xie J, Zhang W, Bian K, Liang J, et al. Differentiating high-grade patterns and predominant subtypes for IASLC grading in invasive pulmonary adenocarcinoma using radiomics and clinical-semantic features. Cancer Imaging. 2025;25(1):42. 40155960 10.1186/s40644-025-00864-2 PMC11951669

[22] Hu M, Zhang J, Cheng Q, Wei W, Liu Y, Li J, et al. Multi-DECT image-based intratumoral and peritumoral radiomics for preoperative prediction of muscle invasion in bladder cancer. Acad Radiol. 2025;32(1):287–97. 39168722 10.1016/j.acra.2024.08.010

[23] Zhou Y, Su G-Y, Hu H, Tao X-W, Ge Y-Q, Si Y, Shen M-P, Xu X-Q, Wu F-Y. Radiomics from primary tumor on Dual-Energy CT derived iodine maps can predict cervical lymph node metastasis in papillary thyroid cancer. Acad Radiol. 2022;29:S222–31. 34366279 10.1016/j.acra.2021.06.014

[24] Cortellini A, Santo V, Brunetti L, Garbo E, Pinato DJ, La Cava G, et al. Transformer-based AI approach to unravel long-term, time-dependent prognostic complexity in patients with advanced NSCLC and PD-L1 ≥ 50%: insights from the pembrolizumab 5-year global registry. J Immunother Cancer. 2025. 10.1136/jitc-2025-012423. 41022528 10.1136/jitc-2025-012423 PMC12481261

[25] Gao S-Y, Zhang X-Y, Wei W, Li X-T, Li Y-L, Xu M, et al. Identification of benign and malignant thyroid nodules by in vivo iodine concentration measurement using single-source dual energy CT. Medicine (Baltimore). 2016. 10.1097/md.0000000000004816. 27684811 10.1097/MD.0000000000004816 PMC5265904

[26] Li Z, Zhong Y, Lv Y, Zheng J, Hu Y, Yang Y, et al. A CT based radiomics analysis to predict the CN0 status of thyroid papillary carcinoma: a two- center study. Cancer Imaging. 2024;24(1):62. 38750551 10.1186/s40644-024-00690-y PMC11094940

[27] Geng D, Zhou Y, Shang T, Su GY, Lin SS, Si Y, et al. Effect of Hashimoto’s thyroiditis on the dual-energy CT quantitative parameters and performance in diagnosing metastatic cervical lymph nodes in patients with papillary thyroid cancer. Cancer Imaging. 2024;24(1):10. 38238870 10.1186/s40644-024-00655-1 PMC10797959

[28] Zhou Y, Su GY, Hu H, Tao XW, Ge YQ, Si Y, et al. Radiomics from primary tumor on dual-energy CT derived iodine maps can predict cervical lymph node metastasis in papillary thyroid cancer. Acad Radiol. 2022;29(Suppl 3):S222-31. 34366279 10.1016/j.acra.2021.06.014

[29] Xu XQ, Zhou Y, Su GY, Tao XW, Ge YQ, Si Y, et al. Iodine maps from dual-energy CT to predict extrathyroidal extension and recurrence in papillary thyroid cancer based on a radiomics approach. AJNR Am J Neuroradiol. 2022;43(5):748–55. 35422420 10.3174/ajnr.A7484 PMC9089265

[30] Song Z, Liu Q, Huang J, Zhang D, Yu J, Zhou B, et al. The value of machine learning based on spectral CT quantitative parameters in the distinguishing benign from malignant thyroid micro-nodules. BMC Cancer. 2025. 10.1186/s12885-025-14450-z. 40597056 10.1186/s12885-025-14450-z PMC12211486

[31] Geng D, Zhou Y, Shang T, Su G-Y, Lin S-s, Si Y, et al. Effect of Hashimoto’s thyroiditis on the dual-energy CT quantitative parameters and performance in diagnosing metastatic cervical lymph nodes in patients with papillary thyroid cancer. Cancer Imaging. 2024;24(1):10. 38238870 10.1186/s40644-024-00655-1 PMC10797959

[32] Ogata T, Ueguchi T, Yagi M, Yamada S, Tanaka C, Ogihara R, et al. Feasibility and accuracy of relative electron density determined by virtual monochromatic CT value subtraction at two different energies using the gemstone spectral imaging. Radiat Oncol. 2013;8:83. 23570343 10.1186/1748-717X-8-83 PMC3627631

[33] Ozguner O, Dhanantwari A, Halliburton S, Wen G, Utrup S, Jordan D. Objective image characterization of a spectral CT scanner with dual-layer detector. Phys Med Biol. 2018;63(2):025027. 29185436 10.1088/1361-6560/aa9e1b

[34] Al Ajmi E, Forghani B, Reinhold C, Bayat M, Forghani R. Spectral multi-energy CT texture analysis with machine learning for tissue classification: an investigation using classification of benign parotid tumours as a testing paradigm. Eur Radiol. 2018;28(6):2604–11. 29294157 10.1007/s00330-017-5214-0

[35] Su KH, Kuo JW, Jordan DW, Van Hedent S, Klahr P, Wei Z, Al Helo R, Liang F, Qian P, Pereira GC, et al. Machine learning-based dual-energy CT parametric mapping. Phys Med Biol. 2018;63(12):125001. 29787382 10.1088/1361-6560/aac711

