# Supplementary material for: Distinguishing benign from malignant thyroid nodules via virtual biopsy: a study on using quantitative parameters and classical radiomics features from dual-energy CT imaging
Source: BMC Cancer. 2025 Nov 25;25:1823. doi: 10.1186/s12885-025-15261-y (PMC12648871; doi:10.1186/s12885-025-15261-y)
Supplement: Supplementary file 1 — Supplementary Material 1. [file 12885_2025_15261_MOESM1_ESM.docx]

**Hyperparameters of the SVM classifier：**

A Support Vector Machine (SVM) was employed to construct radiomics models. To optimize model performance, hyperparameter tuning was conducted on the training set using grid search with 5-fold cross-validation. The search space encompassed values for the regularization parameter C [0.001, 0.01, 0.1, 1, 10, 100], the RBF kernel coefficient γ [0.0001, 0.001, 0.01, 0.1, 1], and kernel types ['linear', 'rbf']. Model performance was evaluated based on the area under the receiver operating characteristic curve (AUC) derived from cross-validation. The optimal hyperparameter configuration—yielding the highest AUC—consisted of the radial basis function (RBF) kernel with C = 1.0 and γ = 0.01. The final model was trained using this optimized set of hyperparameters.

**Table S1** patient baseline characteristics in training and test cohorts

| **Characteristics** | **Training cohort (n=150)** | |  | **Test cohort(n=65)** | |  | **P value†** |
| --- | --- | --- | --- | --- | --- | --- | --- |
|  | **Benign** | **Malignant** | **P value*** | **Benign** | **Malignant** | **P value*** |  |
|  | **(n=47)** | **(n=103)** |  | **(n=20)** | **(n=45)** |  |  |
| Age (years) | 49.94±12.27 | 44.62±12.67 | 0.017 | 57.10±18.16 | 45.20±12.96 | 0.004 | 0.244 |
| Gender (n (%)) |  |  | 0.854 |  |  | 0.533 | 0.884 |
| Female | 34(72.3) | 73(70.9) |  | 16(80) | 31(68.9) |  |  |
| Male | 13(27.7) | 30(29.1) |  | 4(20) | 14(31.1) |  |  |
| Location |  |  | 0.439 |  |  | 0.776 | 0.432 |
| Left | 19(40.4) | 49(47.6) |  | 9(45) | 18(40) |  |  |
| Right | 27(57.4) | 52(50.5) |  | 10(50) | 25(55.6) |  |  |
| Isthmus | 1(2.1) | 2(1.9) |  | 1(5) | 2(4.4) |  |  |
| Lesion morphology |  |  | ＜0.001 |  |  | 0.043 | 0.488 |
| Regular | 39(83) | 51(49.5) |  | 13(65) | 17(37.8) |  |  |
| Irregular | 8(17) | 52(50.5) |  | 7(35) | 28(62.2) |  |  |
| Cystic change |  |  | ＜0.001 |  |  | 0.010 | 0.527 |
| Absent | 26(55.3) | 93(90.3) |  | 12(60) | 42(93.3) |  |  |
| Present | 21(44.7) | 10(9.7) |  | 8(40) | 3(6.7) |  |  |
| Microcalcification |  |  | ＜0.001 |  |  | 0.013 | 0.963 |
| Absent | 44(93.6) | 64(62.1) |  | 18(90) | 29(64.4) |  |  |
| Present | 3(6.4) | 39(37.9) |  | 2(10) | 16(35.6) |  |  |
| Edge interruption sign |  |  | ＜0.001 |  |  | ＜0.001 | 0.884 |
| Absent | 40(85.1) | 60(58.3) |  | 19(95) | 25(55.6) |  |  |
| Present | 7(14.9) | 43(41.7) |  | 1(5) | 20(44.4) |  |  |
| blur after enhancement |  |  | 0.031 |  |  | 0.013 | 0.719 |
| Absent | 40(85.1) | 72(69.9) |  | 18(90) | 29(64.4) |  |  |
| Present | 7(14.9) | 31(30.1) |  | 2(10) | 16(35.6) |  |  |

*Comparison between benign and malignant thyroid nodules. †Comparison between training and test cohorts.

**

**

**Figure. S1** The specific features selected for the 40 keV, 70 keV, 100 keV, IMD, and multi-image models.


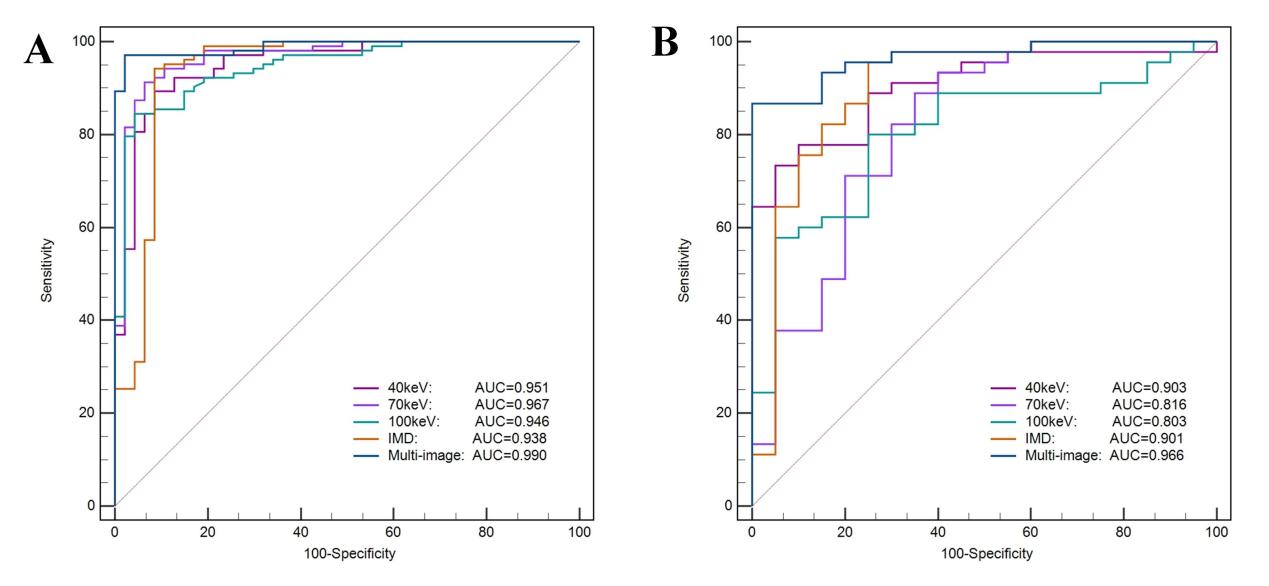


**Figure. S2** Receiver operating characteristic curves for radiomics models in training (**A**) and test cohorts (**B**).


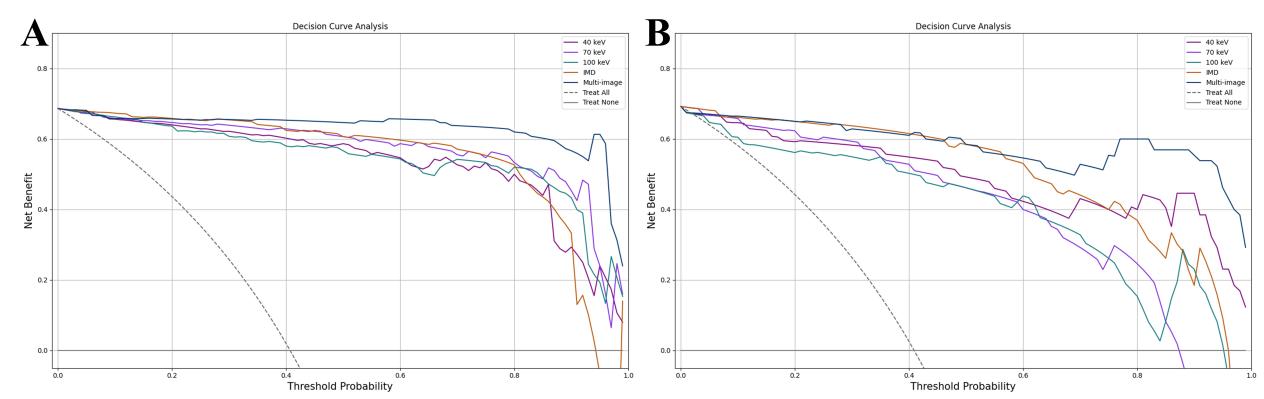


**Figure. S3** Decision curves for radiomics models in training (**A**) and test cohorts (**B**).

**Table S2** The final features selected for the multi-image model

|  | **Image** | **Phase** | **Filter** | **Feature class** | **Feature** |
| --- | --- | --- | --- | --- | --- |
| 1 | IMD | arterial | original | shape | Sphericity |
| 2 | 40 | arterial | wavelet | glszm | wavelet-LLH-GrayLevelNonUniformityNormalized |
| 3 | 40 | venous | wavelet | ngtdm | wavelet-LLH-Contrast |
| 4 | IMD | venous | wavelet | glcm | wavelet-LHL-JointEntropy |
| 5 | 70 | venous | log | glcm | log-sigma-0-5-mm-3D-Imc1 |
| 6 | IMD | venous | log | glszm | log-sigma-4-0-mm-3D-SizeZoneNonUniformityNormalized |
| 7 | 40 | arterial | wavelet | glszm | wavelet-HHH-SizeZoneNonUniformity |
| 8 | 40 | arterial | wavelet | glszm | wavelet-HHH-LowGrayLevelZoneEmphasis |
| 9 | 40 | venous | wavelet | gldm | wavelet-HHH-SmallDependenceHighGrayLevelEmphasis |
| 10 | 70 | venous | log | firstorder | log-sigma-0-5-mm-3D-90Percentile |
| 11 | 70 | venous | log | glcm | log-sigma-1-0-mm-3D-Idn |
| 12 | 70 | venous | log | glcm | log-sigma-1-0-mm-3D-Idm |
| 13 | 40 | venous | wavelet | gIrlm | wavelet-LLH-GrayLevelNonUniformityNormalized |
| 14 | 70 | venous | original | glszm | SmallAreaLowGrayLevelEmphasis |
| 15 | IMD | venous | wavelet | glszm | wavelet-LLL-GrayLevelNonUniformityNormalized |
| 16 | IMD | venous | log | glszm | log-sigma-4-0-mm-3D-SmallAreaEmphasis |
| 17 | 40 | arterial | wavelet | gldm | wavelet-HHL-LargeDependenceEmphasis |
| 18 | IMD | venous | log | glszm | log-sigma-0-5-mm-3D-GrayLevelNonUniformityNormalized |
| 19 | IMD | venous | wavelet | glcm | wavelet-LHH-JointAverage |
| 20 | 40 | venous | wavelet | ngtdm | wavelet-HLH-Busyness |
| 21 | IMD | venous | wavelet | gldm | wavelet-LLH-LargeDependenceLowGrayLevelEmphasis |
| 22 | 100 | arterial | wavelet | ngtdm | wavelet-HHL-Contrast |
| 23 | 100 | arterial | wavelet | ngtdm | wavelet-HHL-Complexity |
| 24 | 70 | arterial | wavelet | glrlm | wavelet-HLL-LongRunHighGrayLevelEmphasis |
| 25 | 70 | venous | original | firstorder | 10Percentile |
| 26 | 70 | arterial | wavelet | glrlm | wavelet-HLL-GrayLevelNonUniformityNormalized |
| 27 | IMD | venous | log | glszm | log-sigma-4-0-mm-3D-SmallAreaHighGrayLevelEmphasis |
| 28 | 100 | arterial | wavelet | ngtdm | wavelet-LLL-Busyness |
| 29 | 40 | arterial | wavelet | gldm | wavelet-LLL-LargeDependenceLowGrayLevelEmphasis |
| 30 | 70 | venous | original | shape | Elongation |
| 31 | IMD | venous | log | glszm | log-sigma-4-0-mm-3D-LargeAreaLowGrayLevelEmphasis |
| 32 | IMD | arterial | original | glcm | Contrast |
